# Adaptive Support Weight-Based Stereo Matching with Iterative Disparity Refinement

**DOI:** 10.3390/s25134124

**Published:** 2025-07-02

**Authors:** Alexander Richter, Till Steinmann, Andreas Reichenbach, Stefan J. Rupitsch

**Affiliations:** 1 Electrical Instrumentation and Embedded Systems, Department of Microsystems Engineering, Albert–Ludwigs–Universität Freiburg, Georges-Köhler-Allee 106, 79110 Freiburg, Germany; till.steinmann@imtek.uni-freiburg.de (T.S.); stefan.rupitsch@imtek.uni-freiburg.de (S.J.R.); 2 Fraunhofer Institute for High-Speed Dynamics, Ernst–Mach–Institut (EMI), Ernst-Zermelo-Straße 4, 79104 Freiburg, Germany

**Keywords:** Computer Vision, minimally invasive surgery, medical endoscopy, real-time 3D reconstruction

## Abstract

Real-time 3D reconstruction in minimally invasive surgery improves depth perception and supports intraoperative decision-making and navigation. However, endoscopic imaging presents significant challenges, such as specular reflections, low-texture surfaces, and tissue deformation. We present a novel, deterministic and iterative stereo-matching method based on adaptive support weights that is tailored to these constraints. The algorithm is implemented in CUDA and C++ to enable real-time performance. We evaluated our method on the Stereo Correspondence and Reconstruction of Endoscopic Data (SCARED) dataset and a custom synthetic dataset using the mean absolute error (MAE), root mean square error (RMSE), and frame rate as metrics. On SCARED datasets 8 and 9, our method achieves MAEs of 3.79 mm and 3.61 mm, achieving 24.9 FPS on a system with an AMD Ryzen 9 5950X and NVIDIA RTX 3090. To the best of our knowledge, these results are on par with or surpass existing deterministic stereo-matching approaches. On synthetic data, which eliminates real-world imaging errors, the method achieves an MAE of 140.06 μm and an RMSE of 251.9 μm, highlighting its performance ceiling under noise-free, idealized conditions. Our method focuses on single-shot 3D reconstruction as a basis for stereo frame stitching and full-scene modeling. It provides accurate, deterministic, real-time depth estimation under clinically relevant conditions and has the potential to be integrated into surgical navigation, robotic assistance, and augmented reality workflows.

## 1. Introduction

Today, surgeons often rely on Minimally Invasive Surgery (MIS), which offers substantial benefits over traditional surgery. MIS uses small incisions and tiny cameras to provide a magnified view, enabling precise operations. This leads to faster recovery, less pain, lower infection risk, reduced blood loss, and minimal scarring [1,2].

In MIS, visual limitations hinder spatial understanding, increasing mental workload and error risks [3,4]. The use of 3D stereo-endoscopes addresses these issues by providing a 3D view, improving instrument navigation, and reducing accidental tissue damage [5].

While monocular camera setups are possible, stereo imaging offers significant advantages for real-time 3D reconstruction. It enables single-shot depth estimation with greater accuracy by eliminating the need to estimate camera motion or adapt to dynamic baseline changes [6].

Despite advancements, the full potential of 3D depth information remains untapped. Accurate data could enable tasks like point-to-point measurements, collision avoidance, or augmented reality, but its accuracy is essential.

Stereo matching estimates depth from two images by calculating pixel disparities. Local stereo-matching methods use color and intensity within a predefined window, which should only include pixels from one depth level. This can be achieved by adjusting the window size and shape or adaptively weighting pixels. The latter, Adaptive Support Weights (ASW), introduced by Yoon and Kweon in 2005 [7], weights pixels by color similarity and spatial gradients before summing them.

We propose a novel iterative stereo-matching method based on the algorithm by Hosni et al. [8], but tailored to the challenges of medical endoscopy. The proposed deterministic approach enhances depth estimation accuracy and reliability in endoscopic procedures, achieving speed and accuracy comparable to state-of-the-art deep learning-based methods. Our method focuses on accurate single-shot 3D reconstruction, laying the foundation for higher-level applications such as stitching or full 3D scanning of the surgical scene.

The contributions of our method include

- An iterative refinement strategy optimized for video-based stereo correspondence;
- Sub-pixel precision in disparity estimation to enhance depth accuracy;
- Real-time, pixel-level disparity range adjustment for dynamic scene adaptation;
- A high-performance, GPU-accelerated implementation of the box filter.

## 2. State of the Art

Historically, 3D reconstruction algorithms originated in robotic vision, focused on enabling machines to perceive and interact with their environment. This pursuit led to methods like stereo vision, Structure from Motion (SfM), and Simultaneous Localization and Mapping (SLAM), later adopted in domains such as autonomous driving, Augmented Reality (AR), and medical surgery.

Despite advances in 3D reconstruction, real-time 3D reconstruction for medical endoscopy remains under-explored. Few algorithms address the unique challenges of endoscopic procedures, such as constrained environments, reflective surfaces, tissue deformation, and real-time depth estimation. That said, several general-purpose algorithms adapted from other fields have been successfully transferred to the medical domain, with some showing promising results.

Comparing 3D reconstruction methods in MIS is challenging due to the lack of standardized evaluation. Some methods use public datasets, while others rely on custom setups, making direct comparisons unfeasible. Evaluation metrics also vary and can include the mean absolute error (MAE), root mean squared error (RMSE), precision, or percentage deviation. In addition, key details such as hardware, frames per second (FPS), or even image resolution are not always included, further hindering objective comparisons.

In recent years, machine learning (ML) algorithms have attracted attention for their ability to adapt across domains given the necessary training data, as well as their abilities in 3D reconstruction. These methods effectively handle large datasets, enhance image reconstruction accuracy, and minimize noise and artifacts.

Rau et al. [9] trained a conditional generative adversarial network for monocular endoscopic images, using real data and phantom data, as well as synthetic data from the Unity graphics engine. The proposed method was evaluated on synthetic data with an average RMSE of 1.75 mm. However, evaluation on phantom data revealed an average RMSE of 16.55 mm ± 0.81 mm.

As another example, Liu et al. [10] combined SLAM with a learning-based approach to refine the sparse depth map generated from SLAM into a dense depth map. The proposed method was evaluated on a synthetic dataset, which was generated analogously to the learning dataset using VR-Caps, consisting of 123 frames with an image resolution of 320 × 320 pixels, resulting in an RMSE of 0.9375 mm and an MAE of 0.4828 mm.

Despite breakthroughs like Transformers improving scalability [11], strict reliability and predictability standards have limited surgical ML’s adoption. Its ‘black-box’ nature and challenges involving liability, ethics [12], and scarce medical training data compared to fields like autonomous driving [13] further hinder its use. Consequently, as of today, deterministic methods remain the predominant choice in operating rooms.

Richter et al. [14] compare 3D reconstruction algorithms, including deterministic binocular methods. Results vary based on the reconstructed image size, as reported by the original authors. The following highlights the most promising results, combining high-resolution input and frame rate.

An early demonstration of real-time stereo reconstruction in MIS was presented by Stoyanov et al. [15], who developed a system for dense depth estimation during robot-assisted procedures. While historically relevant, the dataset and source code are not available at present, limiting reproducibility.

Hosni et al. [8] applied the ASW weighting method by Yoon and Kweon [7] to calculate disparity maps using C++ and Compute Unified Device Architecture (CUDA). Their implementation uses a guided filter for edge preservation and runtime efficiency, independent of the filter size. At 640 × 480 resolution, the algorithm achieves 17 Hz on an NVIDIA GTX 480, with a 5.55% error on the Middlebury dataset.

Ttofis and Theocharides [16] present their implementation of the ASW algorithm on an Field-Programmable Gate Array (FPGA) to lower the power consumption. This hardware-centric implementation achieves an error value of 6.36% on the Middlebury dataset when its images with a resolution of 1280 × 720 pixels are processed with a frame rate of 60 Hz on the Inrevium Kintex-7 FPGA.

Zeffiro’s stereo pipeline, evaluated on the Stereo Correspondence and Reconstruction of Endoscopic Data (SCARED) dataset [17], processed images at 1280 × 1024 resolution, achieving an MAE of 3.47 mm, placing first in the publication’s ranking. The algorithm’s frame rate was not disclosed. Details of the evaluation script are provided in Section 4.3.

The following sections present our method for real-time 3D reconstruction, designed to meet the unique requirements of endoscopic procedures in MIS.

## 3. Proposed Method

The Adaptive SupportWeights Iterative (ASWI) algorithm addresses the challenges of real-time disparity estimation in minimally invasive surgery. It does so by combining adaptive cost aggregation with an efficient iterative refinement strategy. It builds on the algorithm proposed by Hosni et al. [8], which utilizes guided filtering for cost aggregation. However, it incorporates additional mechanisms to enhance spatial consistency without compromising on real-time capability.

The key innovation of our method is the implementation of an iterative neighborhood-based refinement framework that dynamically adapts the potential disparity range for each pixel. This refinement strategy leverages local context by focusing computation within a disparity neighborhood defined by the disparities of surrounding pixels and temporal coherence by using the previous frame’s disparity map to guide iterative updates in the current frame. Together, these mechanisms significantly reduce the computational load while improving the disparity estimation accuracy. Consequently, the ASWI algorithm is particularly well suited to video sequences and continuous input, where real-time updates with high spatial consistency are required.

The proposed algorithm consists of four main stages: (1) initial cost value calculation using a combined color- and gradient-based metric, (2) edge-aware cost matrix smoothing using a guided filter, (3) postprocessing to enforce consistency and reduce artifacts, and (4) an iterative disparity update mechanism that leverages temporal coherence to reduce the computational load. The following subsections provide a detailed description of each component.

### 3.1. Cost Value Calculation

The algorithm begins by computing an initial matching cost for each pixel position *p* (x,y) based on the differences in color and spatial gradients in the stereo images $I_{\mathrm{left}}$ and $I_{\mathrm{right}}$. Due to the different viewing angles of the stereo cameras onto the same 3D scene, objects in the scene appear shifted in the two resulting images. This shift is denoted as the disparity *d*. The disparity is inversely related to the distance from an object to the camera.

Equations (1) and (2) originate from the cost functions, made popular by the Semiglobal matching (SGM) algorithm by Hirschmüller in 2005 [18]. Here, the per-pixel dissimilarity is computed using both color differences and gradient-based measures to improve robustness under varying illumination conditions. These fundamental equations have since been adopted and extended in a wide range of stereo matching methods [8,19,20,21]. Equation (1) calculates the absolute color difference *M* for the RGB channels *i* of the position *p* of a pixel.

(1) $$M\left(p,d\right)=\sum_{i=1}^{3}\left|I_{\mathrm{left}}^{i}\left(p\right)-I_{\mathrm{right}}^{i}\left(p-d\right)\right|$$

The gradient difference *G* is derived in Equation (2) and uses a 3×3 Sobel filter to capture structural changes in the grayscale version of the stereo images denoted by $I^{g}$. Here, $\nabla_{x}$ denotes the gradient in the x-direction, computed using a Sobel filter.

(2) $$G\left(p,d\right)=\left|\nabla_{x}\left(I_{\mathrm{left}}^{g}\left(p\right)\right)-\nabla_{x}\left(I_{\mathrm{right}}^{g}\left(p-d\right)\right)\right|$$

Following Hosni et al. [8], we limit the dissimilarity of the color and spatial gradients to improve the robustness to changes in illumination. This method is adopted from optical flow estimation to mitigate illumination differences [22,23]. To this end, we compute aggregation costs based on truncated color and gradient values, combined with a balance factor α, which determines the weighting between color and spatial gradients. Based on experimental evaluation, a factor α=0.1 was selected to provide an effective trade-off between the spatial and color modalities, favoring location. The truncation thresholds $T_{M}$ and $T_{G}$ that were introduced by Hosni et al. [8] are used to limit the impact of the color and spatial gradient. To formally express this aggregation strategy, the cost function C(p,d) is shown in Equation (3).

(3) $$C\left(p,d\right)=\alpha \;\cdot \;\min\left(T_{M},M\left(p,d\right)\right)+\left(1-\alpha \right)\;\cdot \;\min\left(T_{G},G\left(p,d\right)\right)$$

The initial per-pixel cost estimations tend to be noisy due to local ambiguities and matching errors, thus requiring a subsequent filtering step to reduce outliers and improve the estimation accuracy.

### 3.2. Cost Matrix Smoothing

To reduce the noise introduced during cost aggregation, we apply the guided filter to generate Adaptive SupportWeights (ASW). Introduced by Yoon and Kweon in 2005 [7], the ASW algorithm constructs a local window around each pixel and computes a weighted sum of all pixels within the window. The weights are determined based on the color similarity and spatial proximity relative to the center pixel. While traditional ASW algorithms typically perform explicit aggregation, this step is equivalent to a smoothing of the cost volume that is edge-preserving. Replacing the bilateral filter with the guided filter maintains edge-aware behavior while significantly reducing the computational cost.

Proposed at the 2010 European Conference on Computer Vision [24], the guided filter is an edge-preserving filter using an input image *P* and an RGB guidance image *I*. The guidance image identifies edges, while the input image is filtered. The filter output *q* at pixel position *p* is defined in Equation (4).

(4) $$q_{p}=\sum_{j}W_{p,j}^{\mathrm{GF}}\left(I\right)\;\cdot \;P_{j}$$

Here, *j* denotes the position of the kernel pixel in the image, and $W_{pj}^{\mathrm{GF}}$ denotes the guided filter kernel (or weights). As shown in Equation (5), this kernel is independent of the input image *P* and is computed based solely on the guidance image [24].

(5) $$W_{p,j}^{\mathrm{GF}}\left(I\right)=\frac{1}{{\left|\omega \right|}^{2}}\sum_{k:\left(p,j\right)\in \omega_{k}}\left(1+\frac{\left(I_{p}-\mu_{k}\right)\left(I_{j}-\mu_{k}\right)}{\sigma_{k}^{2}+\epsilon }\right)$$

The square window $\omega_{k}$ has a radius *r*, with |ω| representing the number of pixels in the window. The mean and variance of the guidance image within the window are $\mu_{k}$ and $\sigma_{k}^{2}$, respectively. To penalize large $W_{pj}^{\mathrm{GF}}\left(I\right)$, a regularization parameter ϵ is introduced. As shown by He et al. [25], the guided filter can be closely modeled using box filters, achieving a runtime of O(n). Our implementation follows the pseudo-code in Algorithm 1.
**Algorithm 1** Guided filter pseudo code, where .∗ operator denotes element-wise matrix multiplication, while / represents inverse matrix multiplication. 1:$mean_{I}=f_{\mathrm{mean}}\left(I\right)$ 2:$corr_{I}=f_{\mathrm{mean}}\left(I\;.*\;I\right)$ 3:$var_{I}=corr_{I}-mean_{I}\;.*\;mean_{I}$ 4:$mean_{p}=f_{\mathrm{mean}}\left(P\right)$ 5:$corr_{\mathrm{IP}}=f_{\mathrm{mean}}\left(I\;.*\;P\right)$ 6:$cov_{\mathrm{IP}}=corr_{\mathrm{IP}}-mean_{I}\;.*\;mean_{P}$ 7:$a=cov_{\mathrm{IP}}\;/\;\left(var_{I}+\epsilon U\right)$ 8:$b=mean_{P}-a\;.*\;mean_{I}$ 9:$mean_{a}=f_{\mathrm{mean}}\left(a\right)$10:$mean_{b}=f_{\mathrm{mean}}\left(b\right)$11:**return**  $q=mean_{a}\;.*\;I+mean_{b}$

The aggregation costs serve as the guided filter’s input image, and the left stereo image acts as the guidance image. For each pixel, the smoothed aggregation costs $C^{\prime }$ are computed for all disparity levels using Equation (6).

(6) $$C^{\prime }\left(p,d\right)=\sum_{j}W_{p,j}^{\mathrm{GF}}\left(I\right)\;\cdot \;C\left(j,d\right)$$

After completing the guided filter computation for all disparities, a winner-takes-all strategy is employed across the disparity levels $\Delta_{d}$ to estimate the final disparity for each pixel, denoted as D(p), in the disparity map *D*, as shown in Equation (7). The disparity range $\Delta_{d}$ is not fixed but dynamically refined through the iterative optimization process described in Section 3.4. This enables the algorithm to focus on the most relevant disparity candidates for each region, reducing the likelihood of outliers and improving the computational efficiency.

(7) $$D\left(p\right)=\underset{d\in \Delta_{d}}{arg min}C^{\prime }\left(p,d\right)$$

To further refine the disparity map *D*, a median filter is applied during the postprocessing stage.

### 3.3. Postprocessing

At the beginning of the postprocessing, a weighted median filter is applied to the right disparity map to reduce outliers and improve the accuracy in the iterative step. Next, a left–right consistency check is performed, marking pixels as inconsistent if values differ between positions *p* in the left and p−d in the right disparity map. Inconsistent pixels are filled with the disparity of the nearest consistent pixel by comparing the closest consistent pixels on either side, replacing the inconsistent pixel with the lower-disparity value. Finally, a weighted median filter, which follows the approach by [8] and is described by Equation (8), is applied to all pixels to reduce stripe-like artifacts and enhance accuracy.

(8) $$W_{p,j}^{\mathrm{BL}}\left(I\right)=\frac{1}{K_{p}}\;\cdot \;exp\left(\frac{{\left|p-j\right|}^{2}}{\sigma_{G}^{2}}\right)exp\left(\frac{{\left|I_{p}-I_{j}\right|}^{2}}{\sigma_{M}^{2}}\right)$$

Here, $K_{p}$ denotes a normalization factor, and $\sigma_{G}$ and $\sigma_{M}$ represent the spatial and color dissimilarity thresholds, respectively. The bilateral filter weights $W_{p,j}^{\mathrm{BL}}$ are used because the guided filter weights are computationally too expensive to achieve real-time performance [8].

Lastly, glare rejection is implemented to address reflective surfaces in MIS that saturate sensor pixels with specular LED light, hindering depth estimation. Saturated pixels are marked as inconsistent, and their disparity is estimated as described above.

### 3.4. Iterative Approach

**Figure 1:**
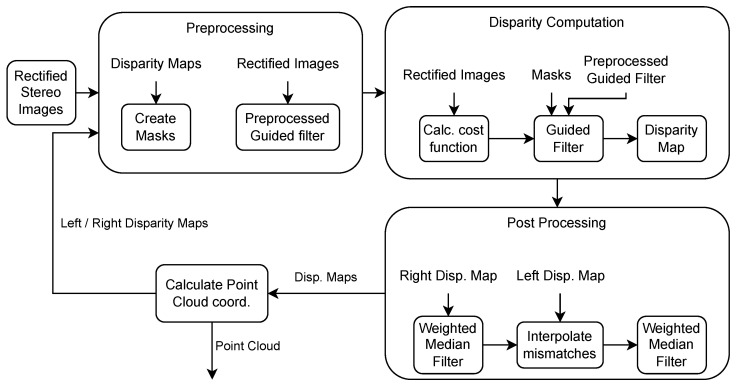
Flowchart of the ASWI algorithm and its iterative approach.

Our key contribution lies in the introduction of an iterative, neighborhood-based optimization that leverages temporal coherence in continuous inputs, such as video sequences. For these types of input, the cost function calculation can be optimized by iteratively updating the disparity values. As shown in Figure 1, this approach uses the previous frame’s disparity map to refine the current one. The cost function and cost matrix smoothing are computed only within a disparity neighborhood $d_{\mathrm{NBH}}$ around pixel position *p* in the disparity map *D*, with an added disparity offset $d_{\mathrm{offset}}$. This neighborhood is defined as $D\left(p\right)\pm d_{\mathrm{offset}}$, where $d_{\mathrm{offset}}$ is an added disparity offset. This neighborhood forms the dynamic disparity range $\Delta_{d}$, which is updated at each iteration to create a mask with minimum and maximum disparity values. Applying this mask reduces the computation range, minimizing outliers and the number of disparities that must be processed.

This optimization not only accelerates processing but also improves disparity map consistency in video sequences. Unlike previous methods that recompute the full cost volume for each frame, our method preserves relevant context while minimizing redundant computation, making it particularly effective for real-time applications.

To account for object motion between frames, we consider the maximum and minimum disparities of neighboring pixels when defining the disparity range. A radius variable extends the mask’s search radius, based on the edge radius, box filter radius, guided filter stage, and the mask’s orientation in the separable convolution. Incorporating this strategy requires careful alignment with the structure of the guided filter.

The guided filter uses the box filter on the guidance image, input image, and their correlations to compute *a* and *b*, as described in Algorithm 1. It then applies the box filter to *a* and *b* to compute the output. Thus, the neighborhood mask size must account for the double-smoothed result and box filter radius.

Separate masks for each stage enable the efficient computation of the guided filter’s two smoothing stages. Each separable convolution stage uses an individual mask denoted as $Mask_{s}$, with *s* being the stage of the guided filter and separable convolution. As stated in Equation (9), allowing iterative disparity updates without evaluating the cost function for all disparities. The cost function is computed only for disparities within the specified range defined by $Mask_{s}\left(p\right)$. Therefore, multiple distinct masks are necessary to accommodate the varying window sizes in the filtering stages of the guided filter, as shown in Equation (10). These masks localize disparity updates for both the guided filter and the cost function.

(9) $${\mathrm{Mask}}_{s}\left(p\right)=\left[\min_{j\in \omega_{r_{s}}\left(p\right)}D\left(j\right)-d_{\mathrm{offset}},\;\max_{j\in \omega_{r_{s}}\left(p\right)}D\left(j\right)+d_{\mathrm{offset}}\right]$$

(10) $$r_{s}\left(x,y\right)=\left\{\begin{array}{cc} \left(e_{r}+b_{r},\;e_{r}+2\;\cdot \;b_{r}\right) & \mathrm{if}\;s=x1\\ \left(e_{r}+b_{r},\;e_{r}+b_{r}\right) & \mathrm{if}\;s=y1\\ \left(e_{r},\;e_{r}+b_{r}\right) & \mathrm{if}\;s=x2\\ \left(e_{r},\;e_{r}\right) & \mathrm{if}\;s=y2 \end{array}\right.$$

Here, D(j) denotes the disparity at pixel position *j* in the disparity map *D*, $e_{r}$ denotes the edge radius, $b_{r}$ denotes the box filter radius, and $\omega_{r_{s}}\left(p\right)$ defines a local window ω with radius $r_{s}$ centered at pixel position *p*. The radius $r_{s}$ has an *x* and *y* component, since the window can be a rectangle, and the parameter $d_{\mathrm{offset}}$ specifies the disparity margin for neighborhood refinement.

In Equation (10), the masks x1, y1, x2, and y2 correspond to the four separable convolution components used to implement the guided filter’s two-stage box filtering. Specifically, x1 and y1 represent the x- and y-directions of the first box filter stage, while x2 and y2 correspond to the second stage. These directional distinctions are necessary because separable convolution treats horizontal and vertical passes independently, each with different smoothing characteristics and window sizes.

The algorithm requires a warm-up period of several frames to fully converge. The edge radius size influences the speed of point cloud conversion. Eliminating the warm-up period accelerates processing but increases outliers in the initial frames, which may be acceptable depending on the application. For benchmarking, the warm-up period was used to achieve optimal results. This approach can also be applied to a single stereo pair to reduce outliers and improve accuracy.

### 3.5. Hardware Optimization

Commonly, a sliding window approach is used to efficiently implement the box filter in the guided filter. Computing the sliding window box filter for a full HD color image on an NVIDIA GeForce GTX 970 GPU (NVIDIA Corporation, Santa Clara, CA, USA) takes approximately 27 ms, which is insufficient when the filter is applied six times per disparity. By using NVIDIA’s shared memory (SM) with separable convolution, the runtime is reduced to one millisecond for a full HD image on a GTX 970. This eliminates the need for the sliding window approach.

SM and separable convolution enable threads to compute a few pixels each, optimizing performance through parallelism. The number of pixels stored in SM during row convolution is given by Equation (11).

(11) $$SM_{size}=\left(pixelsPerThread+2\;\cdot \;padding\right)\;\cdot \;threads$$

**Figure 2:**
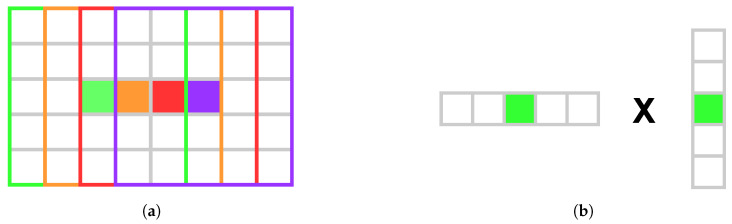
Illustrations of the box filter calculation (**a**) and separable convolution (**b**). (**a**) Shows the box filter with a radius of 2 applied by four threads (green, orange, red, and purple) to four pixels respectively. (**b**) Demonstrates the separable convolution process for the green thread, where parallel computing reduces the runtime complexity from O(x×y) to O(x+y).

Here, threads is the number of threads per block, pixelsPerThread is the number of pixels that each thread processes, and padding ensures an extra window radius to either side of the threads·pixelsPerThread section to ensure that all summations occur in SM, as illustrated in Figure 2a. Figure 2b demonstrates how separable convolution optimizes performance by reducing global memory accesses through SM reuse. Using Equation (11), each thread copies a fixed number of pixels to SM for both rows and columns, improving parallelism by minimizing the workload and leveraging SM’s speed.

## 4. Setup and Evaluation

The ASWI algorithm is evaluated on both the SCARED dataset, which provides varied testing conditions representative of real-world surgical applications, and a synthetic dataset tailored to controlled benchmarking. Medical scenes pose unique challenges for depth estimation, including low color contrast, poor illumination, and specular reflections from wet tissue. Unlike datasets such as Middlebury [26] or KITTI [27], the SCARED dataset replicates these challenges while reflecting the optical characteristics of endoscopic systems, including short working distances and narrow fields of view. However, real-world ground truth is inherently prone to errors. The SCARED dataset, which relies on robot kinematics, exhibits inaccuracies in calibration and temporal alignment. To evaluate our method without these limitations, we developed a synthetic dataset that emulates endoscopic imaging conditions while providing perfect camera poses and noise-free ground truth geometry. While this dataset does not feature photorealistic surgical scenes, it shows simplified geometric shapes and thereby enables controlled benchmarking.

Development and evaluation were conducted on a high-tier consumer-grade system with an AMD Ryzen 9 5950X CPU and an Nvidia RTX 3090 GPU.

### 4.1. Theoretical Approach

In image-based 3D reconstruction algorithms, surface reconstruction error is primarily determined by the resolution of the image acquisition device. Consequently, in stereo-based methods, this resolution limits the accuracy of disparity estimation.

**Figure 3:**
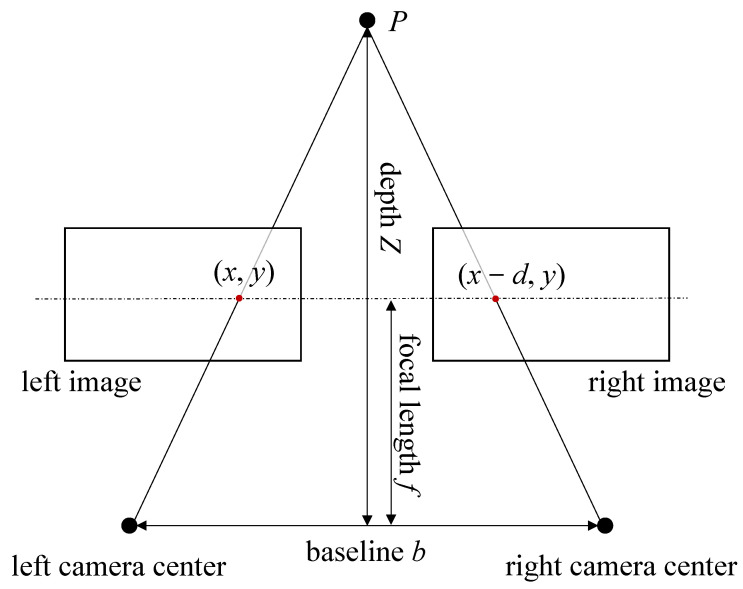
Geometry of a stereo image. The point *P*, located at depth *Z*, is projected onto the image planes of the left and right cameras, which are separated by the baseline *b*. The projected points appear at positions (x,y) in the left image and (x−d,y) in the right image, ideally differing only by the disparity *d* along the horizontal axis in their respective coordinate systems.

When considering a stereo endoscope for medical 3D reconstruction, in analogy to Figure 3, two cameras have circular apertures of diameter *D*, in a scene with light of wavelength λ. The cameras are separated by a distance (baseline) *b*, and they are focused on a point *P* at a distance *Z*, also referred to as depth *Z*, from the aperture plane.

Each camera consists of a lens and an image sensor, both of which can introduce errors into the imaging process. The optical resolution of the lens is ultimately limited by the diffraction per the Rayleigh criterion and chromatic aberration, such as lens imperfections, and the sensor, whose resolution is limited by the number and size of the pixels. Given that these resolutions are smaller, they limit the resolution of the whole system.

The Rayleigh criterion defines the minimum distance at which two points of light can be distinguished as separate [28]. This is particularly relevant for cameras with small apertures, such as those used in medical 3D reconstruction, and it can be used to determine the resolution limit of the imaging system. The resolution of an imaging system can also be limited by optical aberration and or diffraction, causing the image to blur. Aberration describes the unwanted non-Gaussian deviation of light rays through a lens, while diffraction describes the bending or spreading of light rays around obstacles or when passing through small openings. The Rayleigh criterion for all angles is defined by Equation (12).

(12) $$\sin\left(\theta \right)=\frac{1.22\;\cdot \;\lambda }{D}$$

Here, θ is the angular resolution in radians, λ is the wavelength of the light in the scene, and *D* is the diameter of the circular aperture of the camera. That is, two points on the surface that are separated by an angle θ or greater can be resolved by the endoscope camera, while points that are closer together will appear as one. The spatial resolution $d_{RC}$ is the smallest distance between two points that can be detected by the endoscope within the focal plane at a given depth *Z*. The spatial resolution is derived by the tangent of the angular resolution multiplied by the depth *Z*.

For a typical configuration available in medical endoscopy systems, we can assume that *D* = 4.5 mm and *λ* ≈ 650 nm and therefore *θ* ≪ 1. Hence, the Rayleigh criterion and resulting spatial resolution can be simplified using the small-angle approximations sin(θ)≈θ and tan(θ)≈θ, resulting in Equation (13).

(13) $$d_{\mathrm{RC}}=Z\;\cdot \;\tan\left(\theta \right)\approx Z\;\cdot \;\frac{1.22\;\cdot \;\lambda }{D}$$

These parameters refer to the AESCULAP Einstein Vision^®^ PV631 endoscope head. Applying Equation (13) with a common working distance of *Z* = 5 cm yields a theoretical resolution of approximately 8.81 μm.

**Figure 4:**
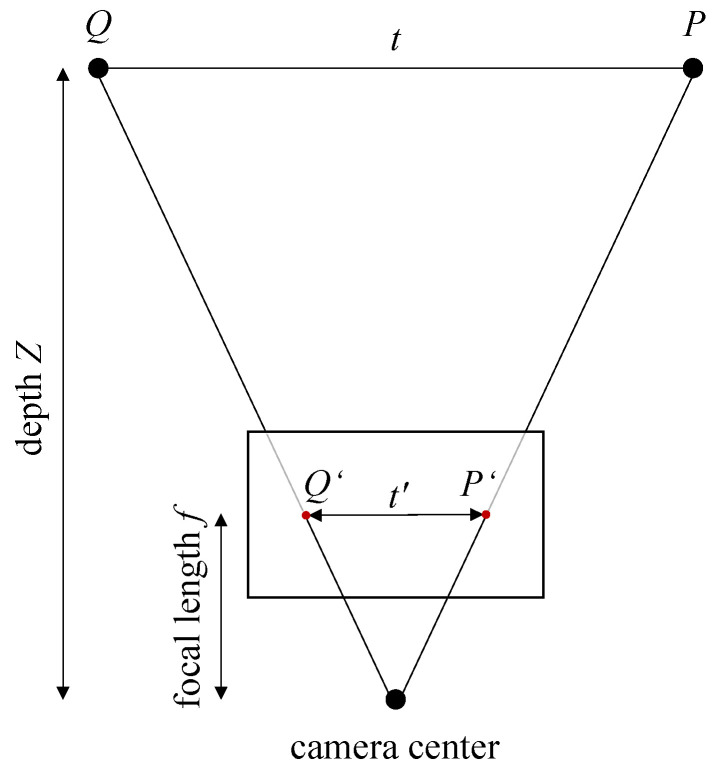
Geometry of the single image based upon [29]. Two points *P* and *Q* are projected onto the image plane as $P^{\prime }$ and $Q^{\prime }$.

To assess the resolution of the image sensor, the geometry of a single image must be considered, as described by Förstner et al. [29] and illustrated in Figure 4.

Two points *P* and *Q* with a distance *t* between them are projected onto the image plane as $P^{\prime }$ and $Q^{\prime }$, with a corresponding image distance $t^{\prime }$. The relationship between *t*, $t^{\prime }$, the focal length *f*, and the working distance (or depth) *Z* is given by Equation (14).

(14) $$\frac{t}{t^{\prime }}=\frac{Z}{f}$$

Assuming square pixels, the smallest structure that can be detected by a sensor is one pixel wide. While sub-pixel accuracy can be achieved by considering the brightness distribution across pixels, a worst-case estimate assumes that an object must be at least the size of a pixel. Then, for a given working distance *Z* and focal length *f*, the width of the smallest resolvable structure in the scene can be calculated using Equation (14). For the AESCULAP Einstein Vision PV631 endoscope (B. Braun Melsungen AG, Melsungen, Germany) used for the SCARED dataset, the product information sheet specifies the sensors as 1/3″, which yields a sensor diagonal of 8.467 mm and a square pixel size of *t*′ = 3.843 μm in consideration of a 16:9 aspect ratio. With a focal length of *f* = 4.62 mm, the structure of size *t* that can be resolved at a given working distance can be calculated using Equation (14) as follows.

(15) $$t=\frac{t^{\prime }\;\cdot \;Z}{f}=\frac{3.84\;\mu m\;\cdot \;5\;cm}{4.62\;mm}\approx 41.56\;\mu m$$

### 4.2. Synthetic Dataset

To evaluate the limits of the algorithm in isolation from real-world imaging errors, we created a synthetic dataset, consisting of camera passes over high-precision-machined test bodies typically used to verify the accuracy of tactile probing systems. This allows for the exclusion of factors such as lens distortion, calibration inaccuracies, sensor noise, and lighting variability. The low-texture test bodies with sharp edges pose challenges intentionally chosen to stress-test the method under idealized yet difficult conditions.

**Figure 5:**
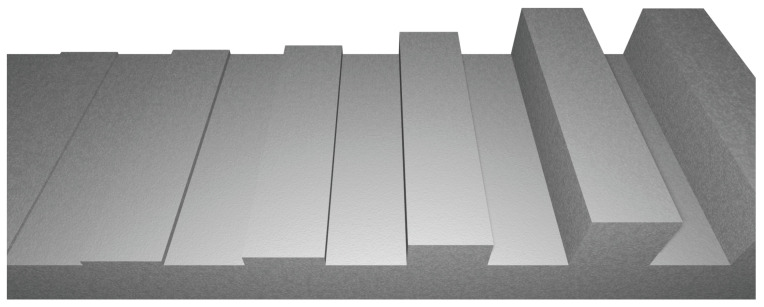
View of a synthetic height profile test body. The profile captures fine surface details and sharp edges under idealized rendering conditions to evaluate algorithm performance in the absence of real-world imaging noise.

The dataset is generated in Blender using a perspective projection stereo camera modeled after a stereo endoscope widely used in clinical procedures, with the key parameters summarized in Table 1. Test bodies are placed in an empty scene with a transparent background, as can be seen in Figure 5. The dataset includes stereo captures of five test bodies from multiple angles, totaling 2500 images. Working distances mimic laparoscopic contexts, measuring 3–12 cm with an average of 5 cm. We intend to make the dataset publicly available as part of a separate publication to support reproducibility and further research.

The rendering setup satisfies the Shannon–Nyquist criterion for the smallest feature sizes, ensuring that fine structures are theoretically resolvable and preventing aliasing artifacts in the generated images. Depth estimation from the ASWI is evaluated via pixel-wise comparison with the ground truth. Since only test bodies are in the scene, the ASWI exclusively reconstructs their surfaces. Ground truth disparity and depth values are obtained using Vision Blender [30]. Regions not visible in both stereo images are excluded from evaluation, as depth cannot be reliably estimated in those areas.

### 4.3. SCARED Dataset

**Figure 6:**
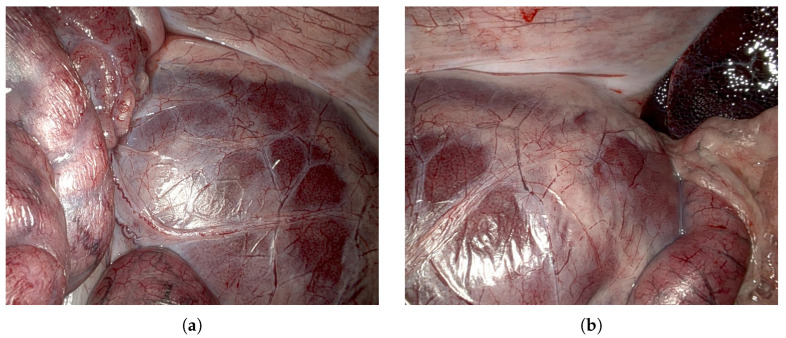
Exemplary RGB frames from the left camera in keyframe 1 of the SCARED dataset 9 [17], illustrating typical scene variation across the sequence. Frame 0 (**a**) and frame 300 (**b**) capture notable changes in viewpoint and organ configuration.

To evaluate the performance of our algorithm under realistic surgical imaging conditions, we use the Stereo Correspondence and Reconstruction of Endoscopic Data (SCARED) dataset, which was released as part of the 2019 Medical Image Computing and Computer Assisted Intervention (MICCAI) Endoscopic Vision Challenge [17]. This benchmark dataset is designed for assessing stereo-matching algorithms in minimally invasive surgery and captures a wide range of anatomical structures and endoscopic viewpoints. It consists of seven training and two test datasets, each recorded on porcine cadavers and containing 4 to 5 keyframes where structured light patterns were projected to generate ground truth depth. Figure 6 shows two representative RGB frames from keyframe 1 in SCARED dataset 9, selected for their range of depth and suitability as realistic examples of endoscopic imaging. The dataset includes an evaluation script that computes the MAE for each keyframe, enabling consistent and reproducible comparisons across algorithms. The authors of the dataset published the performance of ten participating methods in order to provide a reference for comparison. Our results are evaluated alongside several of these in Section 5.

To support accurate 3D reconstruction, the dataset contains detailed stereo calibration files. Table 2 summarizes the intrinsic and extrinsic parameters for both the left and right cameras, including focal lengths, principal points, and distortion coefficients for keyframe 0 in dataset 8. The values were derived from the provided calibration data, and the interocular distance was computed from the stereo baseline translation vector.

**Figure 7:**
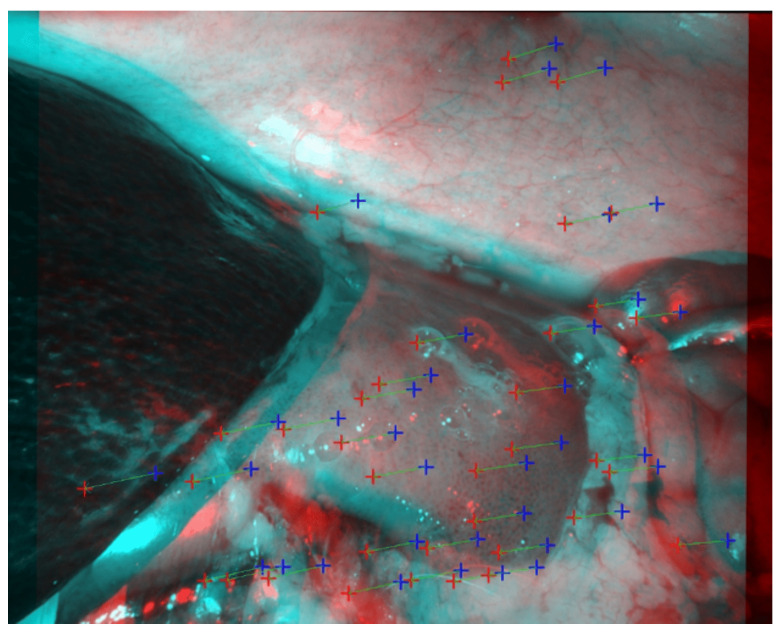
Stereo anaglyph from keyframe 1 of SCARED dataset 4, as published in the original SCARED article [17]. The image shows rectified frames using the provided calibration parameters. Feature correspondences, indicated by red/blue colored crosses connected with green lines, fail to align horizontally, illustrating epipolar geometry violations caused by inaccurate intrinsic calibration.

In the SCARED challenge, the average error for each dataset is computed by first calculating the mean absolute error within each keyframe and then averaging these values with equal weight. This introduces a bias towards keyframes with fewer frames, as each one contributes equally to the overall score regardless of its size. Beyond this, several limitations of the dataset are well documented in the supplementary material of the SCARED journal article [17]. Notably, erroneous calibration files, particularly in datasets 4 and 5, contain intrinsic parameter inaccuracies that result in stereo rectification misalignments, with feature correspondences failing to align along scanlines, as shown in Figure 7. These errors are primarily attributed to flawed intrinsic calibration.

**Figure 8:**
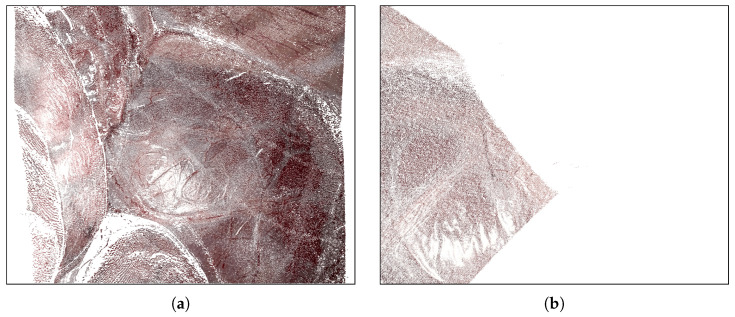
Structured-light ground truth coverage in SCARED dataset 9 [17]. (**a**) Frame 0 from the left camera in keyframe 1 shows dense, uniform coverage, with most of the scene within the structured-light projection volume. (**b**) Frame 300 shows reduced coverage due to camera motion, with large parts of the scene falling out of view. These variations reduce the reliability of depth-based evaluation.

The SCARED dataset, as documented transparently by the authors, exhibits several issues related to ground truth alignment. The RGB video frames and interpolated depth maps, derived from robot kinematics, are not synchronised, leading to misalignment, particularly in interpolation sequences. Moreover, the ground truth format stores 3D coordinates for each pixel in the left image frame, which can result in spatial offsets when reprojected using the provided calibration parameters. These offsets are likely caused by discretisation noise or calibration inaccuracies and are visually apparent in datasets 8 and 9, as illustrated in Figure 8. Several of these problems were independently confirmed by post-challenge participants, including Dimitris Psychogyios, who reported inconsistencies in calibration and rectification. To address such limitations, Schmid and Kurmann proposed a visual reconstruction pipeline that replaces kinematics-based poses with estimates obtained through SIFT feature tracking and perspective-n-point pose estimation. This improves geometric consistency by relying solely on image content.

While these limitations affect the accuracy of the depth annotations, the SCARED dataset remains a valuable benchmark for evaluating stereo-matching algorithms due to its realistic surgical imaging conditions, the availability of precise structured-light-based ground truth, and its widespread adoption in the research community. We therefore benchmark our results against a subset of methods reported in the original SCARED publication [17], as detailed in Section 5.

## 5. Results and Discussion

We evaluated the performance of our algorithm using both a controlled synthetic dataset and real-world surgical data from the SCARED dataset to assess its precision and robustness to real-world acquisition artifacts.

To assess our method’s performance independently of real-world acquisition imperfections, we evaluated our method on a synthetic dataset, as described in Section 4.2. For this, we computed the MAE and RMSE by subtracting the estimated depth map from the ground truth at all non-background pixels within the overlapping field of view of both cameras. The dataset features an average working distance of 53.3 mm, which aligns well with the geometry of typical endoscopic setups. Given the pixel pitch, focal length, and working distance, the resulting resolution meets the sampling spatial sampling requirements necessary to resolve fine surface structures without aliasing. The results obtained on the synthetic dataset are summarized in Table 3, where we compare our method to those by Hosni et al. [8] and Hirschmüller [18]. Our algorithm yields an MAE of 140.06 μm and an RMSE of 251.90 μm and runs at 16.63 FPS, based on averages across the full sequence.

Hosni et al.’s method is based on adaptive support weights and guided filtering, while Hirschmüller’s SGM is a widely used global approach. We evaluated a GPU-accelerated implementation of SGM via the open-source libSGM library by Fixstars Corporation [31], which relies on OpenCV and is limited to a disparity range of 256. To support the larger disparity ranges in our synthetic dataset—similar to those in the SCARED dataset—we also evaluated the CPU-based OpenCV implementation of SGM. This version diverges slightly from the original in its cost metric and reduced path aggregation. While Hosni’s method achieves higher accuracy and SGM offers greater runtime efficiency. Together, they serve as practical benchmarks for evaluating our approach.

**Figure 9:**
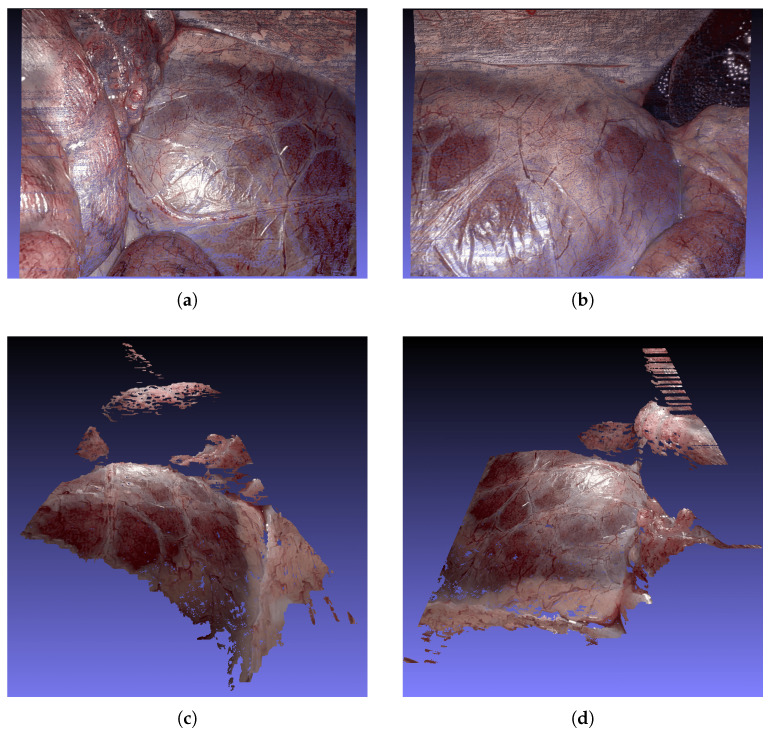
ASWI point cloud reconstructions for keyframe 1 in SCARED dataset 9. (**a**) Top–down view of frame 0. (**b**) Top–down view of frame 300. (**c**) Side view of frame 0. (**d**) Frontal view of frame 0. Together, these perspectives demonstrate consistent geometry and surface continuity.

To assess our method’s performance in real-world medical scenarios, we evaluated our method on the test datasets 8 and 9 of the SCARED benchmark. Figure 9 presents qualitative 3D reconstructions generated by our ASWI algorithm for keyframe 1 of dataset 9. Figure 9a,b show top–down views of Frame 0 and Frame 300, which correspond to the same frames shown in Figure 8. Figure 9c,d provide complementary side and frontal perspectives of Frame 0, offering a more detailed look at the spatial consistency and geometric detail of the reconstructed surfaces.

**Table 3:** Quantitative evaluation on the synthetic dataset, comparing our algorithm to baseline methods by Hirschmüller and Hosni using mean absolute error (MAE), root mean squared error (RMSE), and frames per second (FPS). All values are averaged over the full sequence.

Method	GPU Acc.	MAE	RMSE	FPS
ASWI (ours)	✓	0.14 mm	0.25 mm	16.63
Hosni et al. [8]	✓	1.37 mm	1.65 mm	2.88
Hirschmüller (OpenCV) [18,32]	X	1.46 mm	7.54 mm	3.39
Hirschmüller (libGSM) [18,31]	✓	2.36 mm	8.94 mm	51.05

Consistent with the visual results, Table 4 and Table 5 summarize the quantitative evaluation. Using the official SCARED evaluation script, we computed an MAE of 3.79 mm for dataset 8 and an MAE of 3.61 mm for dataset 9, at a frame rate of 24.9 FPS, averaged across all frames.

**Figure 10:**
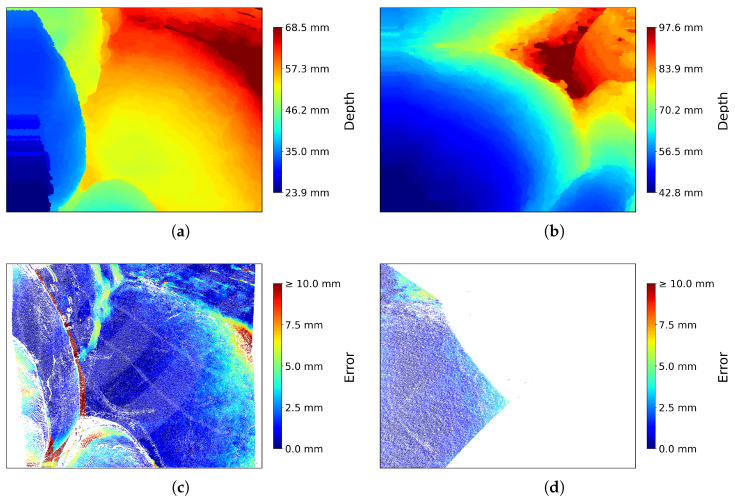
Quantitative visualizations of ASWI depth estimation results for keyframe 1 in SCARED dataset 9. (**a**) Estimated depth map of frame 0. (**b**) Estimated depth map of frame 300. (**c**) Depth error for frame 0, where blue areas indicate alignment with the structured-light ground truth. (**d**) Depth error for frame 300, computed within the valid structured-light region. All error maps follow the official SCARED evaluation protocol.

To further illustrate our method’s performance, Figure 10a,b show the estimated depth maps for Frames 0 and 300, matching the examples used in Figure 8 and Figure 9. The corresponding error maps in Figure 10c,d visualize deviations from the structured-light ground truth. These quantitative visualizations reinforce the spatial consistency observed in the point cloud reconstructions and confirm robustness under real-world conditions.

The significantly lower error observed on the synthetic dataset compared to the SCARED dataset highlights the impact of the data quality on algorithm performance. In the synthetic setting, ground truth depth is perfectly aligned, free from sensor noise, and unaffected by calibration or synchronization artifacts. In contrast, as detailed in Section 4.3, the SCARED dataset includes known sources of error such as inaccurate intrinsic calibration in some sequences, a lack of synchronization between RGB frames and kinematics-based depth maps, and geometric misalignments in datasets 8 and 9 [17]. These factors directly affect the accuracy of any stereo correspondence algorithm and partially explain the higher MAE observed despite the otherwise consistent disparity estimation process. Moreover, the averaging procedure used in the SCARED evaluation protocol, which weighs all keyframes equally regardless of frame count, may further bias the final result. This discrepancy reinforces the importance of using high-quality, well-synchronized ground truth data when benchmarking stereo algorithms and highlights the need for future datasets that ensure both clinical realism and geometric precision.

## 6. Conclusions

We propose a novel deterministic, iterative stereo-matching approach based on the work of Hosni et al. [8], specifically adapted for medical endoscopy. By implementing an iterative strategy, our method enhances accuracy while maintaining the required real-time performance for MIS, achieving 24.9 FPS on consumer hardware from 2020 with CUDA and C++ implementation.

The algorithm was evaluated on both synthetic and real-world datasets. On the SCARED dataset, representing real-world surgical data, it achieved an MAE of 3.79 mm for dataset 8 and 3.61 mm for dataset 9. On synthetic data, free from real-world dataset inaccuracies, it achieved an MAE of 140.06 μm and an RMSE of 251.90 μm. To the best of our knowledge, these results are on par with or surpass existing deterministic stereo-matching approaches. They also demonstrate the algorithm’s ability to deliver reproducible, accurate, real-time 3D depth estimations, demonstrating its suitability for real-world use.

Despite strong performance, challenges remain due to specular reflections that saturate the image sensor. Techniques such as temporal stitching may mitigate artifacts caused by glare but may increase computational demands. However, with its accuracy and real-time performance, the presented algorithm offers potential in applications like robotic-assisted surgery and augmented reality for surgical navigation.

## Figures and Tables

**Table 1:** Camera parameters in Blender based on an Aesculap^®^ Einstein Vision^®^ 3D endoscope.

Parameter	Value
Camera type	Perspective projection
Focal length *f*	4.618 mm
Field of view	72°
Sensor width	6.71 mm
Sensor resolution	1920×1080
Interocular distance	4 mm
Angle to the vertical	135°

**Table 2:** Camera intrinsics and extrinsics for the SCARED dataset 8. Focal lengths and distortion parameters are obtained from the provided calibration data. The interocular distance is derived from the stereo baseline translation vector.

Parameter	Left Camera	Right Camera
Focal length $f_{x}$ (px)	1024.09	1024.20
Focal length $f_{y}$ (px)	1023.89	1023.99
Principal point $c_{x}$ (px)	601.81	696.75
Principal point $c_{y}$ (px)	508.13	507.49
Distortion coefficient $k_{1}$	−2.52 · 10^−3^	−3.26 · 10−3
Distortion coefficient $k_{2}$	+4.39 · 10^−3^	+5.70 · 10^−3^
Distortion coefficient $k_{3}$	+9.22 · 10^−5^	+7.57 · 10^−5^
Image resolution	1280×1024 px	
Interocular distance	4.35 mm	

**Table 4:** Resulting MAE in mm from the evaluation tool for all keyframes of SCARED dataset 8. This table presents a subset of results from the original SCARED publication [17], with the addition of our own ASW and ASWI results for comparison. The ASWI algorithm was configured with a disparity offset of two and an edge radius of seven.

	Key- Frame 0	Key- Frame 1	Key- Frame 2	Key- Frame 3	Key- Frame 4	Average
J.-C. Rosenthal	8.25	3.36	2.21	2.03	1.33	3.44
Trevor Zeffiro	7.91	2.97	1.71	2.52	2.91	3.60
ASWI (Ours)	9.25	2.91	2.21	2.15	2.43	3.79
Congcong Wang	6.30	2.15	3.41	3.86	4.80	4.10
[…]						
Hosni et al. [8]	17.43	12.70	9.50	12.27	12.38	12.86

**Table 5:** Resulting MAE in mm from the evaluation tool for all keyframes of SCARED dataset 9. This table presents a subset of results from the original SCARED publication [17], with the addition of our own ASW and ASWI results for comparison. The ASWI algorithm was configured with a disparity offset of two and an edge radius of seven.

	Key- Frame 0	Key- Frame 1	Key- Frame 2	Key- Frame 3	Key- Frame 4	Average
Trevor Zeffiro	5.59	1.67	4.34	3.18	2.79	3.47
ASWI (Ours)	6.05	1.72	6.79	2.27	1.24	3.61
J.-C. Rosenthal	8.26	2.29	7.04	2.22	0.42	4.05
Congcong Wang	6.57	2.56	6.72	4.34	1.19	4.28
[…]						
Hosni et al. [8]	8.24	11.93	12.60	17.71	3.90	10.88

## Data Availability

The synthetic dataset generated for this study is available upon request from the authors and is planned to be released as part of a separate publication, which will include detailed documentation and access instructions. The SCARED dataset used in the evaluation is available upon request from the organizers of the SCARED challenge via the EndoVis 2019 Grand Challenge platform.
